# PREVENTING ELDER MISTREATMENT THROUGH THE COACH INTERVENTION: WHAT’S RISK GOT TO DO WITH IT?

**DOI:** 10.1093/geroni/igae098.0936

**Published:** 2024-12-31

**Authors:** Kathleen Wilber, Zachary Gassoumis, Eleanor Batista-Malat

**Affiliations:** University of Southern California, Los Angeles, California, United States; University of Southern California, Los Angeles, California, United States; University of Southern California, Los Angeles, California, United States

## Abstract

The Comprehensive Older Adult and Caregiver Help (COACH) program was developed based on effective family-focused approaches from child maltreatment and intimate partner violence. Building on evidence-based prevention strategies, COACH offered from three to 12 sessions with a trained care coach. Sessions used a person-centered strength-based approach designed to help caregivers to reduce stress and burden, seek additional outside support, and better understand and manage the symptoms and experiences of people living with dementia. An evaluation of COACH using a double-blind, randomized controlled trial found a significant reduction in the treatment group compared to no change in the control group. However, no change in risk or protective factors were noted with the exception of social support. This session will explore these findings in relationship to what was learned about risk and protective factors as means to identify and address abuse. Findings suggest that risk and protective factors are helpful to identify and support those at risk of abusing, while other strategies should be considered and tested in developing interventions to prevent and address elder mistreatment.

